# The Black Panther, Masculinity Barriers to Medical Care, and Colorectal Cancer Screening Intention Among Unscreened American Indian/Alaska Native, Black, and White Men

**DOI:** 10.3389/fpubh.2022.814596

**Published:** 2022-04-06

**Authors:** Ellen Brooks, Jessica Y. Islam, David G. Perdue, Ethan Petersen, Marlene Camacho-Rivera, Carson Kennedy, Charles R. Rogers

**Affiliations:** ^1^Department of Family and Preventive Medicine, University of Utah School of Medicine, Salt Lake City, UT, United States; ^2^Center for Immunization and Infections in Cancer, Cancer Epidemiology Program, H. Lee Moffitt Cancer and Research Institute, Tampa, FL, United States; ^3^MNGI Digestive Health, Minneapolis, MN, United States; ^4^Department of Community Health Sciences, SUNY Downstate Health Sciences University, Brooklyn, NY, United States

**Keywords:** health equity (MeSH), Indigenous peoples, men's health (MeSH terms), surveys and questionnaires (MeSH), colonic neoplasms

## Abstract

**Objective:**

To determine if masculinity barriers to medical care and the death from colorectal cancer (CRC) of actor Chadwick Boseman (The Black Panther) influenced CRC early-detection screening intent among unscreened American Indian/Alaska Native (AIAN) and Non-Hispanic-Black (Black) men compared with Non-Hispanic-White (White) men.

**Methods:**

Using a consumer-panel, we surveyed U.S. men aged 18–75 years (*N* = 895) using the 24-item Masculinity Barriers to Medical Care (MBMC) scale. We calculated the median score to create binary exposures to evaluate associations with CRC screening intent and conducted multivariable logistic regression to evaluate independent associations stratified by race/ethnicity.

**Results:**

Overall, Black respondents were most likely to have a high MBMC score (55%) compared to White (44%) and AIAN (51%) men (*p* = 0.043). AIAN men were least likely to report CRC screening intent (51.1%) compared with Black (68%) and White men (64%) (*p* < 0.001). Black men who reported the recent death of Chadwick Boseman increased their awareness of CRC were more likely (78%) to report intention to screen for CRC compared to those who did not (56%) (*p* < 0.001). Black men who exhibited more masculinity-related barriers to care were more likely to intend to screen for CRC (OR: 1.76, 95% CI: 0.98–3.16) than their counterparts, as were Black men who reported no impact of Boseman's death on their CRC awareness (aOR: 2.96, 95% CI: 1.13–7.67). Conversely, among AIAN men, those who exhibited more masculinity-related barriers to care were less likely to have CRC screening intent (aOR: 0.47, 95% CI: 0.27–0.82) compared with their counterparts.

**Conclusions:**

Masculinity barriers to medical care play a significant role in intention to screen for CRC. While Black men were most likely to state that The Black Panther's death increased their awareness of CRC, it did not appear to modify the role of masculine barriers in CRC screening intention as expected. Further research is warranted to better understand how masculine barriers combined with celebrity-driven health-promotion interventions influence the uptake of early-detection screening for CRC.

**Impact:**

Our study provides formative data to develop behavioral interventions focused on improving CRC screening completion among diverse men.

## Introduction

Colorectal cancer (CRC) is the second most common cause of cancer-related death among both men and women in the United States and is characterized by significant and persistent disparities among stigmatized communities (1–10). For example, for all sexes combined, non-Hispanic-African-American/Black (Black) and American Indian/Alaska Native (AIAN) adults experience higher CRC incidence (45.7 and 43.3 per 100,000, respectively) and mortality (19.0 and 15.8 per 100,000, respectively) than Non-Hispanic Whites (Whites) (2, 3, 7, 11). CRC incidence has been found to be 24–27% higher (11) and mortality 36–52% higher among Black men compared with White men (8, 11, 12). Black adults have the lowest rate of 5-year CRC survival across all racial, ethnic, and sex subgroups (11, 13, 14). AIAN males have higher CRC prevalence at all subsites and are the only racial subgroup not experiencing improvements in CRC survival (2, 8, 13). Moreover, cancer disparities in AIAN populations may be underestimated due to racial misclassification and other errors in registries and public records (15, 16).

Although early-detection screening and increasingly effective therapies have significantly decreased CRC incidence and mortality among adults over age 50 years (2, 8, 11, 17–19), the incidence of and mortality from early-onset CRC, which occurs in individuals under age 50 years, have been increasing for at least 3 decades (2, 8, 20, 21). Early-onset CRC incidence is twice as high among Black adults as among White adults (3). Between 1973 and 1994, Blacks were diagnosed with early-onset CRC at a rate 53.4% higher than that for Whites (22). Rates for Blacks remained higher than those for Whites through the mid-2000s, creating a widening racial gap (2). From 2012 to 2016, early-onset CRC incidence increased by 2% among Whites and by 2.2% among AIANs (2, 23).

In 2018 the American Cancer Society endorsed a reduction from 50 to 45 years in the recommended age at which average-risk adults should initiate CRC screening (24); the U.S. Preventive Services Task Force endorsed a similar recommendation in May 2021 (25). As of 2016, CRC screening rates for adults aged 50 years and older were 68%, 65%, and 59% for Whites, Blacks, and AIANs, respectively (2). Insights are needed to inform CRC screening-uptake strategies, specifically in the context of Black and AIAN men, who experience both the highest rates of CRC in the United States and the most significant barriers to equitable screening uptake.

Among U.S. men, and particularly among Black men, intent to receive CRC screening is often negatively influenced by an endorsement of traditional hegemonic masculinity (i.e., stereotypical, society-driven male traits), which places a high value on identifying as strong, independent, and self-reliant (14, 26, 27). Men have also exhibited reluctance to seek preventive health services for fear of showing weakness and vulnerability (26, 28, 29). Evidence also shows that men may delay or avoid endoscopic CRC screenings as such examinations may expose a sense of vulnerability or elicit fears related to sexual orientation (30, 31). Heslin et al. (32) found that men who identify as gay or bisexual were 67% more likely to have completed CRC screening than those who identified as heterosexual. These findings demonstrate the complex interactions among masculine role norms, intention to undergo invasive screening tests, and fears of being perceived as gay (31).

Masculinity beliefs have also been found positively correlated with medical mistrust barriers to CRC screening among Black men (29). Valuing traditional hegemonic masculinity increases the likelihood of delaying medical care, thereby reducing the frequency of contact with the health-care system and impeding patient-provider trust building (29, 33, 34). Prior experiences of discrimination may influence perceptions of future discrimination in health care (29); medical mistrust is higher when Black men expect to be treated differently because of their race (29, 35). Both historical and present-day instances of racial profiling and fatal forms of racism (e.g., police brutality) provide context for the medical mistrust exhibited by Black men (29, 34, 36). Historical trauma, or collective trauma experienced by groups that share a history of oppression or victimization spanning generations, and conflicting views of healing also underpin AIAN mistrust of Western medicine by inciting suspicion and concern due to unethical treatment (37–41). However, few studies have examined low CRC screening rates among Black and AIAN men in the context of medical discrimination, access to care, and CRC-related beliefs and perceptions (42–46), and limited research has examined these low rates within the framework of masculinity.

The importance of CRC screening and its relationship to masculinity barriers to care attracted attention with the death in August 2020 of the Black actor Chadwick Boseman, which surprised many because of Boseman's family's decision to keep his early-onset CRC diagnosis private (47–49). Best known for his title role in the 2018 movie *Black Panther*, Boseman was diagnosed with stage III early-onset CRC in 2017, which progressed to stage IV and led to his death at age 43 (49). Although many people had questions and concerns about CRC after Boseman's death (47), the impact of this event on CRC screening behaviors among U.S. men is unknown.

Given current trends and disparities in CRC incidence among Black, AIAN, and White men, examination of inhibitive masculinity beliefs that affect CRC screening behavior is warranted to close the intention-behavior gap (3, 14, 22, 26, 27, 50). Further, if celebrity presence influences health behaviors (51, 52), Boseman's death from early-onset CRC may be associated with men's CRC screening intent. The purpose of this study was to determine if masculinity barriers to medical care influenced CRC early-detection screening intent among unscreened AIAN and Black men compared with their White counterparts. Our secondary objective was to evaluate if the death of Chadwick Boseman (The Black Panther) played a role in intention to be screened for CRC. Our hypothesis was that masculinity barriers to medical care would be associated with decreased CRC screening intent. We further hypothesized that self-reported awareness of the death of The Black Panther would positively modify the association of masculinity barriers and intention to screen for CRC.

## Materials and Methods

### Data Source and Collection

The study protocol was approved by the University of Utah Institutional Review Board (IRB #00113679) prior to data collection. From November 2020 to January 2021, we partnered with Qualtrics (Provo, UT), a research software and commercial survey sampling company, to recruit a diverse convenience sample of men from sources such as targeted email lists, customer-loyalty web portals, and social media. Men who (a) were aged 18–75 years; (b) self-described as Black, AIAN, or White; (c) resided in the United States; and (d) understood English were eligible to participate. Those who participated in the survey—that took up to 15 mins to complete—were invited to choose a method of compensation (e.g., points toward retail purchases, frequent flyer miles).

### Measures

Our main exposure was a summary score to estimate masculinity barriers, which was measured using a previously developed survey instrument: the Masculinity Barriers to Medical Care (MBMC) scale validated by Rogers et al. (53). The MBMC scale consists of six theoretically derived factors (subscales) or psychological determinants of men's health on CRC screening completion: (1) Provider Role; (2) Health-related Self-Reliance; (3) Health Problem Minimization; (4) Restrictive Emotionality; (5) Fear of being Perceived as Gay; and (6) Medical Mistrust. For all subscales, individual items were assessed on a 5-point Likert-type scale ranging from 1 (Not at all true) to 5 (Completely True). Each scale item has been listed in Supplementary Table 1. We also used a modified version of the Male Role Norms, Knowledge, Attitudes, and Perceptions associated with CRC Screening instrument validated by Rogers, Goodson, and Obidike to evaluate multi-faceted factors additional to barriers potentially stemmed from masculinity that may influence CRC screening behaviors (54). Through this modified instrument we included two scales: knowledge about CRC and early detection screening (21 true/false items) and Beliefs and Values about CRC and early detection screening (54 items on a 5-point Likert-type scale ranging from 1 or strongly disagree to 5 or strongly agree). We added the question “Did the recent death of Chadwick Boseman (The Black Panther) increase your awareness of colorectal cancer?,” providing three answer choices (Yes, No, Unsure).

Our main outcome was intention to screen for CRC. This was measures using the following question: “Do you plan to obtain colorectal cancer screening in the future?” The answer options were: (1) Yes, in the next 6 months; (2) Yes, in 7 months to 1 year; (3) Yes, in 13 months to 2 years; (4) Yes, sometime, but not within 2 years; (5) No, but have considered getting screened; and (6) No, will not get screened.” Participants were categorized as having intent to screen if they chose options one through three. Participants younger than 45 years (outside of the CRC screening recommendation age group), were categorized as having intent to screen if they picked option four.

### Data Analyses

Descriptive univariate analyses were conducted using Chi-square tests to evaluate the sociodemographic characteristics of our study population. Similarly, MBMC scale items were descriptively presented overall and by racial/ethnic categories. Variations in responses to each scale item by racial and ethnic category were also evaluated using Chi-square tests. For each participant, we calculated an overall MBMC score and a score for each MBMC subscale (Provider Role, Health-Related Self Reliance, Health Problem Minimization, Restrictive Emotionality, Fear of Being Perceived as Gay, and Medical Mistrust) (53).

To compare the MBMC scores across racial/ethnic categories and conduct multivariable analyses, we dichotomized the main exposure and subscale scores using the study population's median score as the categorization definition cut-off. Participants with a score below the study-population median were categorized as exposed and others as unexposed; more-negative composite scores indicated more masculinity-related barriers to care. Given the MBMC score was not our main outcome, we evaluated the functional form of our continuous exposure and determined the most interpretable and transportable function of our main exposure would be categorical (55).

Our outcome was intention to screen for CRC (yes/no) as described above. Multivariable logistic regression was used to calculate adjusted odds ratios (aOR) with 95% confidence intervals (95% CI) to evaluate the association of CRC screening intent with the presence of significant masculinity barriers to medical care as measured by the MBMC scale overall and for each subscale. We estimated these associations stratified by racial/ethnic categories to present specific estimates for each group. Based on the literature and our previous research (14, 54, 56, 57), each model was adjusted for age, marital status, educational attainment, insurance status, having a regular medical-care provider, and family history of CRC. We assessed each covariate for collinearity and used a complete case approach due to limited missing data of our covariates (<2%). For each model, we conducted a stratified multivariable analysis to evaluate the potential modifying effect of Boseman's death on CRC awareness. Given the recently updated guidelines recommending the initiation of CRC screening at age 45 years (25), we performed a sensitivity analysis, evaluating the MBMC items descriptively and repeating the multivariable analyses among participants aged 45 years and older. Due to sample-size limitations within each racial/ethnic category we could not evaluate the impact of Boseman's death on CRC awareness in this sensitivity analysis. The Type I error was maintained at 5%. Based on the exploratory nature of this analysis, we did not include an adjustment for multiple comparisons (58, 59). All statistical analyses were conducted a priori and used Stata IC 15 (StataCorp LLC, College Station, TX).

## Results

Table 1 presents the sociodemographic characteristics of our study population (*N* = 895) overall and stratified by racial and ethnic categories. Age categories were evenly distributed overall and among AIAN adults; however, Black respondents were generally younger (38% aged 18–29 years) and White respondents generally older (9.2% aged 18 to 29 years) (*p* < 0.001). Sixty-two percent of White men were married compared with 49% of AIAN men and 39% of Black men (*p* < 0.01). Nearly half of White men had a bachelor's degree or above, whereas 38% and 42% of Black and AIAN men, respectively, reported a high school education or below (*p* < 0.001). Nearly one-third of AIAN respondents reported daily tobacco use compared with 28% of Black and 27% of White respondents (*p* = 0.005). Black respondents were more likely than Whites or AIANs to be health professionals (24%, 15%, and 14% respectively; *p* = 0.001). AIAN men were less likely than Black or White men to report having a regular medical-care provider (63%, 70%, and 82%, respectively; *p* < 0.001). About three-quarters of participants reported seeing a provider in the last 12 months. Nine and four percent of respondents, respectively, reported a family history or a personal diagnosis of CRC. Black men were more likely (58%) than White (34%) or AIAN men (25%) to report that Boseman's death increased their CRC awareness (*p* < 0.001). Supplementary Table 1 summarizes the distribution of each item in the MBMC scale overall and by racial and ethnic categories.

**Table 1:** Sociodemographic characteristics of 895 diverse men to evaluate masculinity barriers and intention to screen for CRC (November 2020 to January 2021).

			**Race and ethnicity**						
	**Total**		**Black**		**White**		**AIAN**		***P* value**
	**No**.	**Col%**	**No**.	**Col%**	**No**.	**Col%**	**No**.	**Col%**	
**Age group**									**<0.001**
18–29	215	24.0	116	38.3	28	9.2	71	24.8	
30–44	245	27.4	88	29.0	78	25.5	79	27.6	
45–59	215	24.0	46	15.2	104	34.0	65	22.7	
60–75	220	24.6	53	17.5	96	31.4	71	24.8	
**Census region**									**<0.001**
South	231	25.9	89	29.4	73	23.9	69	24.3	
West	330	37.0	108	35.6	97	31.7	125	44.0	
Northeast	90	10.1	28	9.2	35	11.4	27	9.5	
Midwest	185	20.7	68	22.4	67	21.9	50	17.6	
DC and Puerto Rico	57	6.4	10	3.3	34	11.1	13	4.6	
**Marital status**									**<0.001**
Single	337	37.7	154	50.8	77	25.2	106	37.3	
Married/In a relationship	448	50.2	118	38.9	192	62.7	138	48.6	
Divorced/widowed/separated	108	12.1	31	10.2	37	12.1	40	14.1	
**Sexual orientation**									**0.024**
Heterosexual	817	91.5	288	95.0	280	91.5	249	87.7	
Homosexual	32	3.6	8	2.6	11	3.6	13	4.6	
Bisexual/questioning	44	4.9	7	2.3	15	4.9	22	7.7	
**Educational attainment**									**<0.001**
High school or below	296	33.1	115	38.0	62	20.3	119	41.9	
Some college/associates degree	293	32.8	102	33.7	103	33.7	88	31.0	
Bachelor's degree and above	304	34.0	86	28.4	141	46.1	77	27.1	
**Health professional**									**0.001**
No	735	82.3	230	75.9	260	85.0	245	86.3	
Yes	158	17.7	73	24.1	46	15.0	39	13.7	
**Income category**									**<0.001**
34,999 and below	393	44.1	141	46.7	89	29.1	163	**57.4**	
35,000–74,999	276	30.9	103	34.1	91	29.7	82	**28.9**	
75,000 and above	223	25.0	58	19.2	126	41.2	39	**13.7**	
**Employed**									**<0.001**
No	202	22.6	82	27.2	44	14.4	76	26.8	
Yes	690	77.4	220	72.8	262	85.6	208	73.2	
**Insurance status**									**<0.001**
No	175	19.6	60	19.9	36	11.8	79	27.8	
Yes	717	80.4	242	80.1	270	88.2	205	72.2	
**Religious affiliation**									**<0.001**
Christian	561	62.9	212	70.2	207	67.6	142	50.0	
Muslim	19	2.1	8	2.6	5	1.6	6	2.1	
Atheist	63	7.1	12	4.0	34	11.1	17	6.0	
Other	249	27.9	70	23.2	60	19.6	119	41.9	
**Frequency of religious activity attendance**									**0.004**
Never	315	35.3	88	29.1	113	36.9	114	40.1	
Occasionally	381	42.7	142	47.0	115	37.6	124	43.7	
Regular	196	22.0	72	23.8	78	25.5	46	16.2	
**Current tobacco smoking frequency**									**0.005**
Daily	259	29.0	84	27.8	82	26.8	93	32.7	
Less than daily	99	11.1	42	13.9	22	7.2	35	12.3	
Not at all	516	57.8	169	56.0	200	65.4	147	51.8	
Don't know	18	2.0	7	2.3	2	0.7	9	3.2	
**Has regular provider**									**<0.001**
No	250	28.0	90	29.8	54	17.6	106	37.3	
Yes	642	72.0	212	70.2	252	82.4	178	62.7	
**Has seen provider in last 12 mos**									**0.042**
No	225	25.2	76	25.2	64	20.9	85	29.9	
Yes	667	74.8	226	74.8	242	79.1	199	70.1	
**Family history of cancer**									**0.005**
No	513	57.5	191	63.2	168	54.9	154	54.2	
Yes	290	32.5	80	26.5	117	38.2	93	32.7	
Not sure	89	10.0	31	10.3	21	6.9	37	13.0	
**Family history of CRC**									**0.059**
No	686	76.9	234	77.5	241	78.8	211	74.3	
Yes	80	9.0	28	9.3	32	10.5	20	7.0	
Not sure	126	14.1	40	13.2	33	10.8	53	18.7	
**Ever diagnosed with CRC**									**0.247**
No	861	96.5	288	95.4	295	96.4	278	97.9	
Yes	31	3.5	14	4.6	11	3.6	6	2.1	
**Did the recent death of Chadwick Boseman increase your awareness of CRC?**									**<0.001**
No	543	60.9	127	42.1	203	66.3	213	75.0	
Yes	349	39.1	175	57.9	103	33.7	71	25.0	

**Figure 1:**
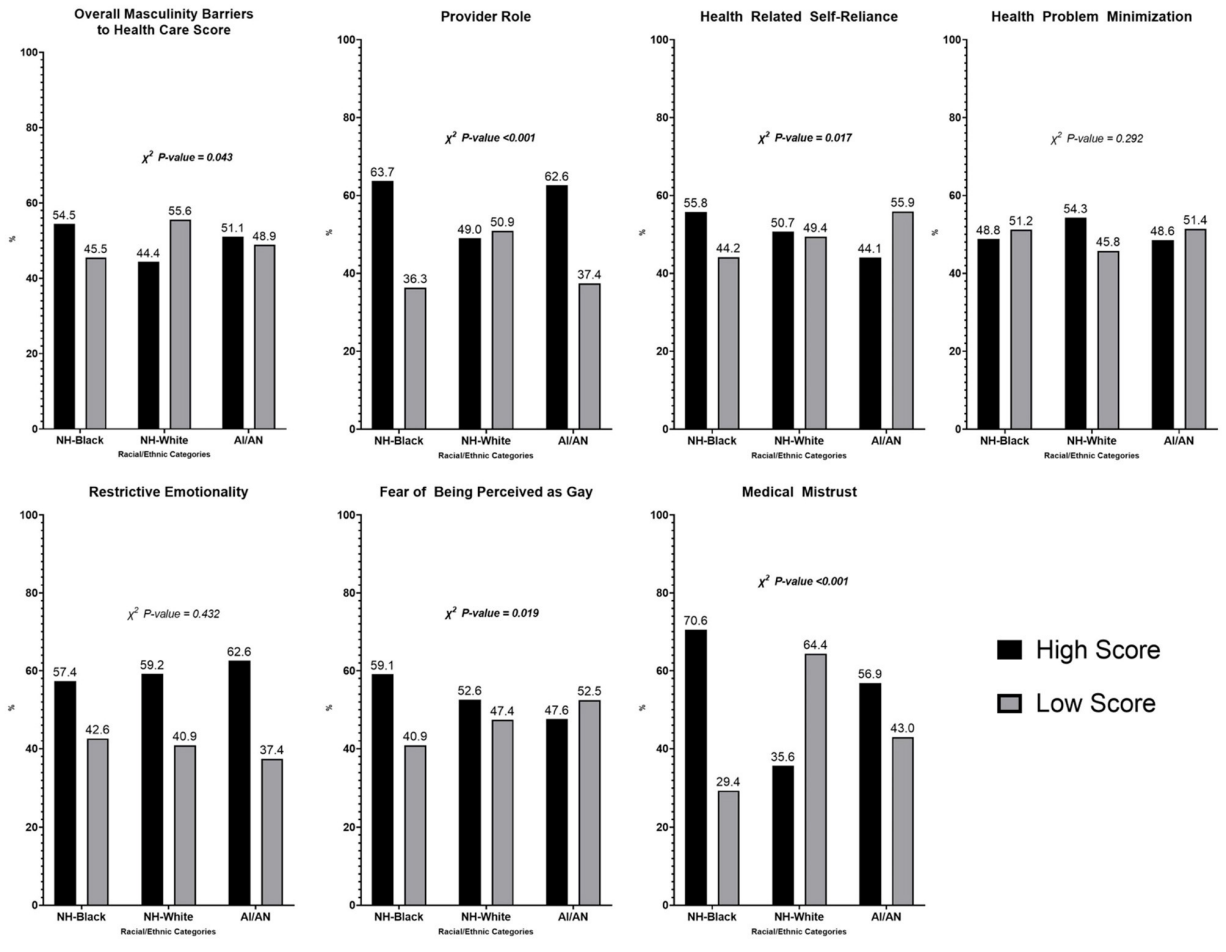
Proportion of high/low scores of overall MBMC and sub-scale specific compared by race/ethnicity. High/low scores defined based on the study population's median score.

Figure 1 compares high and low scores on the overall MBMC scale and each subscale by racial and ethnic categories. Fifty-four percent of Black men had high masculinity-barrier scores compared with 51% of AIAN and 44% of White men (*p* = 0.043). Black (64%) and AIAN men (63%) both had higher scores on the Provider Role subscale than White men (49%) (*p* < 0.001). Black men had higher scores or were more likely to exhibit masculinity barriers in the context of fear of being perceived as gay (59%) compared with White (53%) and AIAN (48%) men (*p* = 0.019). Black men were also more likely to exhibit medical mistrust as a masculinity barrier to care (71%) compared with AIAN (57%) and White (36%) men (*p* < 0.001).

Sixty-two percent of participants reported intent to be screened for CRC. Black men who reported the recent death of Chadwick Boseman (i.e., The Black Panther) increased their awareness of CRC were more likely (78%) to report intention to screen for CRC compared to those who did (56%) (*p* < 0.001). Similar differences were observed among White men (*p* < 0.001), however, not among AIAN men (*p* = 0.064), suggesting the death of The Black Panther did not influence AIAN men. AIAN men were also least likely to report CRC screening intent (51.1%) compared with Black (68%) and White men (64%) (*p* < 0.001).

Overall, significant differences were observed in the role of masculinity barriers to care in CRC screening intent (Table 2). Black men who exhibited more masculinity-related barriers to care were more likely to have CRC screening intent (OR: 1.76, 95% CI: 0.98–3.16) than their counterparts, as were Black men who reported no impact of Boseman's death on their CRC awareness (aOR: 2.96, 95% CI: 1.13–7.67). Conversely, among AIAN men, those who exhibited more masculinity-related barriers to care were less likely to have CRC screening intent (aOR: 0.47, 95% CI: 0.27–0.82) compared with their counterparts; this observation persisted among AIAN men aged 45 years and above (Supplementary Table 3).

**Table 2:** Associations of masculinity barriers to health care with intention to screen for CRC among racial ethnic minority men.

	**Black adult men**			**White adult men**			**AIAN adult men**	
	**OR**	**95% CI**		**OR**	**95% CI**		**OR**	**95% CI**	
**Overall scale score**									
**Overall**	**1.76**	**0.98**	**3.16**	**0.72**	**0.42**	**1.25**	**0.47**	**0.27**	**0.82**
Black Panther increased CRC awareness	1.34	0.57	3.12	2.56	0.55	11.90	0.76	0.19	3.13
No impact of Black Panther on CRC awareness	2.96	1.13	7.67	0.61	0.32	1.15	0.42	0.22	0.79
**Provider role**									
**Overall**	**1.08**	**0.62**	**1.88**	**0.76**	**0.46**	**1.27**	**0.77**	**0.45**	**1.33**
Black Panther increased CRC awareness	1.00	0.44	2.29	0.73	0.22	2.47	0.76	0.20	2.87
No impact of Black Panther on CRC awareness	1.16	0.50	2.69	0.93	0.50	1.72	0.73	0.39	1.39
**Health-related self-reliance**									
**Overall**	**1.44**	**0.85**	**2.55**	**1.55**	**0.95**	**2.55**	**1.01**	**0.59**	**1.72**
Black Panther increased CRC awareness	2.54	1.11	5.81	1.62	0.52	5.03	0.52	0.13	2.08
No impact of Black Panther on CRC awareness	0.95	0.41	2.17	1.49	0.82	2.68	1.09	0.60	1.99
**Health problem minimization**									
**Overall**	**0.82**	**0.47**	**1.41**	**0.68**	**0.40**	**1.14**	**0.52**	**0.30**	**0.92**
Black Panther increased CRC awareness	0.55	0.25	1.26	0.66	0.19	2.22	1.32	0.33	5.32
No impact of Black Panther on CRC awareness	1.37	0.58	3.12	0.82	0.44	1.51	0.42	0.21	0.83
**Restrictive emotionality**									
**Overall**	**0.79**	**0.46**	**1.35**	**0.53**	**0.32**	**0.89**	**0.52**	**0.31**	**0.89**
Black Panther increased CRC awareness	0.84	0.38	1.84	1.10	0.35	3.49	0.82	0.25	2.71
No impact of Black Panther on CRC awareness	0.70	0.31	1.59	0.44	0.27	0.84	0.46	0.24	0.88
**Fear of being perceived as gay**									
**Overall**	**1.55**	**0.89**	**2.71**	**0.79**	**0.47**	**1.34**	**1.06**	**0.62**	**1.82**
Black Panther increased CRC awareness	1.52	0.68	3.37	0.93	0.26	3.34	1.41	0.42	4.79
No impact of Black Panther on CRC awareness	2.13	0.86	5.27	0.71	0.39	1.32	1.02	0.54	1.93
**Medical mistrust**									
**Overall**	**1.56**	**0.88**	**2.78**	**0.64**	**0.37**	**1.10**	**1.14**	**0.66**	**1.96**
Black Panther increased CRC awareness	1.57	0.66	3.78	1.79	0.43	7.42	1.72	0.45	6.54
No impact of Black Panther on CRC awareness	1.36	0.59	3.13	0.57	0.30	1.08	1.04	0.55	1.95

*Adjusted for age, marital status, educational attainment, insurance status, regular provider, family history of CRC*.

Among Black men who reported that Boseman's death increased their CRC awareness, those who exhibited health-related self-reliance as a masculinity barrier were more likely to report CRC screening intent (aOR: 2.54, 95% CI: 1.11–5.81). Among AIAN men, those who exhibited health-problem minimization (aOR: 0.52, 95% CI: 0.30–0.92) and restrictive emotionality (aOR: 0.52, 95% CI: 0.31–0.89) as masculinity barriers were less likely to report CRC screening intent. These associations persisted among AIAN men who reported no impact of Boseman's death on their CRC awareness (Table 2).

## Discussion

CRC screening continues to be underutilized by U.S. men, particularly among racial and ethnic minorities, due to several well-documented barriers (60–64). We evaluated by racial and ethnic categories the role of masculinity barriers to medical care in the context of CRC screening intent. We also probed the potential impact of the August 2020 death from early-onset CRC of Chadwick Boseman, the Black actor best known for portraying The Black Panther, as a modifier of CRC screening intent. Overall, we observed that Black and AIAN men exhibited more masculinity barriers to care than White men, specifically in the context of medical mistrust and their provider role, while Black men were more likely than White or AIAN men to report that Boseman's death increased their CRC awareness.

Our hypothesis that higher masculinity barriers to care would be associated with decreased CRC screening intent was upheld for AIAN men, while among Black men we saw the reverse association. This finding contrasts with prior literature that underscores a direct relationship between masculinity barriers and reduced intention to undergo medical care among Black men (14, 27). One possible reason for our counterintuitive finding is that Black study participants were generally younger than White and AIAN participants; over 67% of Black participants were aged 18–44 compared to 34% of White and 52% of AIAN men. Boseman's death at age 43—which occurred less than 3 months before we began data collection—may have indirectly influenced younger Black men in our sample, leading them to disregard masculine barriers typically observed in that cohort.

It is well established, however, that intentions do not always translate into behavior (65). Moreover, while Boseman's death may have increased CRC awareness among Black men, it is unclear whether increased awareness alone translates to increased CRC screening uptake. A celebrity's announcement of a cancer diagnosis has been shown to produce heightened interest in that cancer. The most well-known case is the so-called “Angelina Effect,” a term coined by *Time* magazine to describe the impact of the actress Angelina Jolie's risk-reducing bilateral mastectomy in 2013 (66). A 2016 analysis, however, revealed that although rates of genetic testing for breast cancer increased following the disclosure of Jolie's procedure, the rate of mastectomies among those tested declined (67). Perhaps even more well known, and pertinent, was the “Katie Couric Effect” which was tied to a dramatic increase in CRC screening (52). This suggests that heightened awareness alone may be insufficient to increase the completion rate of early-detection screening for CRC.

A previous study (68) of the impact of Boseman's death on web-based search interest in CRC found that relative search volume (RSV) for the topics *colorectal cancer* and *colon cancer screening* increased by 598 and 707%, respectively, and were on average 121 and 256% greater than expected during the first 3 months following Boseman's death. The race or ethnicity of those conducting these searches could not be ascertained due to the infoveillance nature of the study; however, during the 2 weeks following Boseman's death, statistically significant positive correlations, which persisted for 4 months, were seen between the RSV for *colon cancer* and the percentage of Black Americans per state and per metropolitan area. All considered, while celebrity messages related to cancer prevention and screening may provide important opportunities to increase awareness of a specific cancer (69), these events may have differential impacts across diverse racial and ethnic groups in public health campaigns tailored to health-disparity populations.

In our current study, among Black men who reported that Boseman's death increased their CRC awareness, high health-related self-reliance was positively associated with CRC screening intent. Self-reliance emphasizes autonomy and independent decision-making, which may motivate engagement in preventive health behaviors that are conducive to self-management (70, 71). However, some studies have found that masculine beliefs, including belief in self-reliance, do not accurately predict CRC screening outcomes and may in fact lead to decreased engagement with preventive health behaviors (8, 26–28). Further research is warranted to better understand how conceptions of masculine self-reliance influence CRC screening-uptake behaviors among men and the impact of celebrity-driven health promotion interventions on CRC early-detection screening completion.

To our knowledge, this is the first study to explore the relationship between masculinity and CRC screening intent in AIAN men. Overall, AIAN men who experienced more masculinity barriers to care were less likely to report CRC screening intent. This association persisted in AIAN men aged 45 years and older and in those who reported no impact of Boseman's death on their CRC awareness. That Boseman's death appeared to have less impact on CRC awareness and screening intent among AIAN men compared with Black men highlights the complex intersection of masculinity and race/ethnicity. Boseman, despite his celebrity status, was not AIAN and therefore his death likely failed to resonate as a cautionary tale with many AIAN men.

Previous work on barriers to CRC screening suggests a need to culturally tailor messages and media to specific AIAN tribal groups to optimize efficacy (72–74). Low CRC knowledge and perceived susceptibility, combined with cultural taboos around discussing certain body parts, fatalistic ideologies, and mistrust of the healthcare system have previously been found to influence CRC screening behaviors among AIAN men (74). Context and culture are critical factors affecting masculinity barriers between populations. Pride, independence, and privacy surrounding one's health have been shown to be negatively associated with CRC screening uptake in AIAN populations; these values are more common among rural AIAN people, especially those dwelling on reservations (70). Mistrust of Western medicine, including negative impressions of clinical and cultural competence, combined with cultural beliefs (e.g., CRC is “a death sentence,” talking about cancer is taboo) are commonly cited barriers to CRC screening among AIAN populations (74). Prior studies have found that higher levels of traditionalism, including the use of traditional healers and fatalistic health beliefs, are associated with lower acceptance and utilization of Western medicine, including colonoscopy (6, 46). Further investigation of the relationship between masculinity, AIAN traditionalism, and CRC screening intent is warranted to inform collaboration with AIAN health systems and address persistent disparities in CRC screening and outcomes in this population.

In our study, high restrictive emotionality and health problem minimization scores were negatively associated with CRC screening intent among AIAN men, paralleling our observations in Black and White men. This concordance suggests these measures may be less race or ethnic specific but rather common traits in men who forgo preventive health care. CRC screening, especially colonoscopy, requires men to be vulnerable and to believe vulnerability to be worth the potential benefit. This sense of vulnerability may elicit fears related to sexual orientation and may compound the reasons for delaying preventive screenings (30, 31). Fear of embarrassment has been found to be a barrier to CRC screening in AIAN men (72). Men with high restrictive emotionality may struggle with the vulnerability requirement, while those who minimize the seriousness of CRC may see screening as an unnecessary inconvenience (75, 76).

To contextualize our findings, several limitations of our methodological approach should be considered. First, as the survey was cross-sectional, we cannot ascertain the temporality of self-reported barriers to care and CRC screening intent. Our study sample was comprised of a convenience sample of respondents to an online survey, which limits the generalizability of our findings. Additionally, there is a potential for selection bias given that only those with access to the internet or broadband were able to participate. Future research should focus on rural populations with potentially limited broadband access or limited use of electronic platforms. We did not include Hispanic/Latino men in the current assessment as the three racial groups we examined have the highest incidence and mortality rates for CRC among male populations, however, have future projects underway to evaluate both masculinity barriers and levels of machismo as they relate to CRC screening history and intention.

Additionally, several limitations relate to the MBMC scale as it undergoes further authentication and optimization. First, some statements may have been vague and resulted in misinterpretation. For example, the statement “When it comes to my health, I rely on the opinions of others” is loaded with two positive medical-professional–specific items (on factor 4 in the four-factor version), suggesting respondents may have thought that “others” could include medical professionals. Next, since 38% of our sample were single, some of the men may have felt they did not have a provider role making the MBMC-related questions less relevant for this specific construct. Additionally, some scale items were double-barreled (two questions in one), which may have resulted in suboptimal participant responses to the Provider Role subscale. Nonetheless, our study is novel and provides unique insights into the potential influence of masculinity on CRC screening intent among a diverse sample of men.

In conclusion, our survey-based study of a diverse cohort of U.S. men aged 18–75 years identified greater masculinity barriers to medical care, particularly relating to medical mistrust and the provider role, among Black and AIAN men than among their White counterparts. Black men were more likely than White or AIAN men to report that the death from early-onset CRC of The Black Panther lead actor Chadwick Boseman increased their CRC awareness. While celebrity messages around cancer prevention and screening may provide important opportunities to increase CRC awareness, these events may have differential impacts across diverse racial/ethnic groups when public health campaigns are tailored to health-disparity populations.

This study additionally demonstrates the need for further research regarding the effects of masculinity barriers to care on CRC screening to ultimately close the intentional-behavior gap. Our findings can inform future multi-level interventions to ensure optimal uptake of CRC screening through optimized patient-provider communication.

## Data Availability Statement

The raw data supporting the conclusions of this article will be made available by the authors, without undue reservation, upon request.

## Ethics Statement

The studies involving human participants were reviewed and approved by University of Utah IRB. The patients/participants provided their written informed consent to participate in this study.

## Author Contributions

CRR: conception, design, and study supervision. CRR, JI, and C-R: development of methodology. JI: analysis and interpretation of data (e.g., statistical analysis, biostatistics, and computational analysis). CRR, JI, EB, EP, DP, CK, and C-R: writing, review, and/or revision of the manuscript. All authors contributed to the article and approved the submitted version.

## Funding

This research was supported by 5 For the Fight and the Huntsman Cancer Institute, the V Foundation for Cancer Research, and the National Cancer Institute (Grant K01CA234319), an entity of the National Institutes of Health (NIH).

## Author Disclaimer

The content is solely the responsibility of the authors and does not necessarily represent the official views of the NIH, 5 For the Fight, V Foundation for Cancer Research, Huntsman Cancer Institute, or the University of Utah.

## Conflict of Interest

Although unrelated to this study, CRR offers scientific input to research studies through an investigator services agreement between the University of Utah and Exact Sciences. The remaining authors declare that the research was conducted in the absence of any commercial or financial relationships that could be construed as a potential conflict of interest.

## Publisher's Note

All claims expressed in this article are solely those of the authors and do not necessarily represent those of their affiliated organizations, or those of the publisher, the editors and the reviewers. Any product that may be evaluated in this article, or claim that may be made by its manufacturer, is not guaranteed or endorsed by the publisher.
