# Supplementary material for: The Black Panther, Masculinity Barriers to Medical Care, and Colorectal Cancer Screening Intention Among Unscreened American Indian/Alaska Native, Black, and White Men
Source: Front Public Health. 2022 Apr 6;10:814596. doi: 10.3389/fpubh.2022.814596 (PMC9019156; doi:10.3389/fpubh.2022.814596)
Supplement: Supplementary file 1 [file Data_Sheet_1.pdf]

Supplementary Table 1: Masculinity barriers to medical care scale measures by race/ethnicity (n=891)

	**Total**		**Black**		**White**		**AIAN**		
	**No.**	**Col %**	**No.**	**Col %**	**No.**	**Col %**	**No.**	**Col %**	**P-Value**
**As a provider, I assure the needs of my family are met.**									**0.039**
Not at all	37	4.2	15	5.0	7	2.3	15	5.3	
Slightly True	66	7.4	31	10.3	19	6.2	16	5.6	
Moderately True	129	14.5	50	16.6	46	15.0	33	11.6	
Mostly True	191	21.4	51	16.9	72	23.5	68	23.9	
Completely True	468	52.5	154	51.2	162	52.9	152	53.5	
**As a provider, I take risks for my family even if I may get hurt or put myself in danger.**									**0.002**
Not at all	134	15.0	48	15.9	49	16.0	37	13.0	
Slightly True	112	12.6	36	12.0	43	14.1	33	11.6	
Moderately True	189	21.2	61	20.3	77	25.2	51	18.0	
Mostly True	169	19.0	56	18.6	67	21.9	46	16.2	
Completely True	287	32.2	100	33.2	70	22.9	117	41.2	
**As a provider, I have been influenced by male family members to be active in my family's life.**									**0.111**
Not at all	158	17.7	52	17.3	48	15.7	58	20.4	
Slightly True	95	10.7	39	13.0	24	7.8	32	11.3	
Moderately True	189	21.2	63	20.9	68	22.2	58	20.4	
Mostly True	206	23.1	66	21.9	87	28.4	53	18.7	
Completely True	243	27.3	81	26.9	79	25.8	83	29.2	
**As a provider, I get an extra push to succeed for my family when I believe somebody is expecting me to fail.**									**0.007**
Not at all	146	16.4	40	13.3	53	17.3	53	18.7	
Slightly True	82	9.2	27	9.0	31	10.1	24	8.5	
Moderately True	207	23.2	72	23.9	79	25.8	56	19.7	
Mostly True	192	21.5	63	20.9	78	25.5	51	18.0	
Completely True	264	29.6	99	32.9	65	21.2	100	35.2	

**When it comes to my health, I figure problems out on my own.**									**0.041**
Not at all	95	10.7	38	12.6	25	8.2	32	11.3	
Slightly True	164	18.4	58	19.3	57	18.6	49	17.3	
Moderately True	250	28.1	74	24.6	105	34.3	71	25.0	
Mostly True	232	26.0	78	25.9	81	26.5	73	25.7	
Completely True	150	16.8	53	17.6	38	12.4	59	20.8	
**When it comes to my health, I rely on the opinions of others.**									**0.001**
Not at all	230	25.8	95	31.6	57	18.6	78	27.5	
Slightly True	205	23.0	59	19.6	71	23.2	75	26.4	
Moderately True	244	27.4	66	21.9	99	32.4	79	27.8	
Mostly True	132	14.8	52	17.3	50	16.3	30	10.6	
Completely True	80	9.0	29	9.6	29	9.5	22	7.7	
**When it comes to my health, I make my own decisions.**									**0.001**
Not at all	39	4.4	24	8.0	5	1.6	10	3.5	
Slightly True	86	9.7	35	11.6	28	9.2	23	8.1	
Moderately True	203	22.8	76	25.2	67	21.9	60	21.1	
Mostly True	244	27.4	77	25.6	95	31.0	72	25.4	
Completely True	319	35.8	89	29.6	111	36.3	119	41.9	
**When it comes to my health, I rely on remedies that others suggest to me.**									**0.087**
Not at all	198	22.2	78	25.9	60	19.6	60	21.1	
Slightly True	225	25.3	62	20.6	78	25.5	85	29.9	
Moderately True	247	27.7	76	25.2	94	30.7	77	27.1	
Mostly True	138	15.5	49	16.3	47	15.4	42	14.8	
Completely True	83	9.3	36	12.0	27	8.8	20	7.0	
**A problem with my health would be a big deal.**									**<0.001**
Not at all	52	5.8	14	4.7	8	2.6	30	10.6	
Slightly True	101	11.3	23	7.6	36	11.8	42	14.8	
Moderately True	219	24.6	68	22.6	82	26.8	69	24.3	

Mostly True	219	24.6	69	22.9	90	29.4	60	21.1	
Completely True	300	33.7	127	42.2	90	29.4	83	29.2	
**I wait until I am sure a problem with my health is serious.**									**0.04**
Not at all	183	20.5	71	23.6	48	15.7	64	22.5	
Slightly True	169	19.0	56	18.6	58	19.0	55	19.4	
Moderately True	237	26.6	77	25.6	88	28.8	72	25.4	
Mostly True	184	20.7	60	19.9	78	25.5	46	16.2	
Completely True	118	13.2	37	12.3	34	11.1	47	16.5	
**I take action immediately when I have a health problem.**									**<0.001**
Not at all	78	8.8	16	5.3	24	7.8	38	13.4	
Slightly True	171	19.2	42	14.0	70	22.9	59	20.8	
Moderately True	222	24.9	73	24.3	89	29.1	60	21.1	
Mostly True	197	22.1	75	24.9	67	21.9	55	19.4	
Completely True	223	25.0	95	31.6	56	18.3	72	25.4	
**A problem with my health will go away on its own.**									**0.009**
Not at all	332	37.3	104	34.6	108	35.3	120	42.3	
Slightly True	200	22.4	65	21.6	73	23.9	62	21.8	
Moderately True	194	21.8	55	18.3	77	25.2	62	21.8	
Mostly True	109	12.2	50	16.6	35	11.4	24	8.5	
Completely True	56	6.3	27	9.0	13	4.2	16	5.6	
**It is important to talk to others about my feelings.**									**0.018**
Not at all	124	13.9	33	11.0	37	12.1	54	19.0	
Slightly True	168	18.9	54	17.9	66	21.6	48	16.9	
Moderately True	255	28.6	85	28.2	84	27.5	86	30.3	
Mostly True	197	22.1	73	24.3	77	25.2	47	16.5	
Completely True	147	16.5	56	18.6	42	13.7	49	17.3	
**I believe in remaining strong at all times.**									**0.008**
Not at all	36	4.0	12	4.0	10	3.3	14	4.9	

Slightly True	80	9.0	35	11.6	30	9.8	15	5.3	
Moderately True	196	22.0	53	17.6	81	26.5	62	21.8	
Mostly True	261	29.3	85	28.2	97	31.7	79	27.8	
Completely True	318	35.7	116	38.5	88	28.8	114	40.1	
**Acknowledging my own emotions is always helpful.**									**0.001**
Not at all	65	7.3	17	5.6	15	4.9	33	11.6	
Slightly True	125	14.0	35	11.6	49	16.0	41	14.4	
Moderately True	209	23.5	65	21.6	84	27.5	60	21.1	
Mostly True	245	27.5	85	28.2	93	30.4	67	23.6	
Completely True	247	27.7	99	32.9	65	21.2	83	29.2	
**Physical pain in my body would never lead me to show any emotions.**									**0.048**
Not at all	207	23.2	75	24.9	80	26.1	52	18.3	
Slightly True	181	20.3	55	18.3	65	21.2	61	21.5	
Moderately True	234	26.3	74	24.6	90	29.4	70	24.6	
Mostly True	158	17.7	58	19.3	43	14.1	57	20.1	
Completely True	111	12.5	39	13.0	28	9.2	44	15.5	
**Having medical exams below the waist would disrespect my sexuality.**									**<0.001**
Not at all	589	66.1	166	55.1	210	68.6	213	75	
Slightly True	73	8.2	29	9.6	26	8.5	18	6.3	
Moderately True	120	13.5	46	15.3	40	13.1	34	12	
Mostly True	65	7.3	34	11.3	23	7.5	8	2.8	
Completely True	44	4.9	26	8.6	7	2.3	11	3.9	
**Medical professionals touching me below the waist is fine when it relates to my health.**									**0.012**
Not at all	57	6.4	26	8.6	12	3.9	19	6.7	
Slightly True	72	8.1	30	10.0	19	6.2	23	8.1	
Moderately True	147	16.5	53	17.6	49	16.0	45	15.8	
Mostly True	177	19.9	63	20.9	73	23.9	41	14.4	
Completely True	438	49.2	129	42.9	153	50.0	156	54.9	

**Health problems below the waist would be embarrassing to me.**									**0.056**
Not at all	291	32.7	94	31.2	87	28.4	110	38.7	
Slightly True	162	18.2	54	17.9	57	18.6	51	18	
Moderately True	188	21.1	61	20.3	76	24.8	51	18	
Mostly True	136	15.3	44	14.6	55	18.0	37	13	
Completely True	114	12.8	48	15.9	31	10.1	35	12.3	
**I would be comfortable with others knowing about health problems below the waist.**									**0.989**
Not at all	247	27.7	84	27.9	84	27.5	79	27.8	
Slightly True	148	16.6	45	15.0	51	16.7	52	18.3	
Moderately True	216	24.2	77	25.6	74	24.2	65	22.9	
Mostly True	148	16.6	50	16.6	53	17.3	45	15.8	
Completely True	132	14.8	45	15.0	44	14.4	43	15.1	
**Medical professionals want men of my race to live a long time.**									**<0.001**
Not at all	97	10.9	38	12.6	19	6.2	40	14.1	
Slightly True	97	10.9	51	16.9	17	5.6	29	10.2	
Moderately True	261	29.3	83	27.6	89	29.1	89	31.3	
Mostly True	211	23.7	70	23.3	83	27.1	58	20.4	
Completely True	225	25.3	59	19.6	98	32.0	68	23.9	
**Men of my race are treated like guinea pigs by medical professionals.**									**<0.001**
Not at all	358	40.2	75	24.9	170	55.6	113	39.8	
Slightly True	162	18.2	60	19.9	41	13.4	61	21.5	
Moderately True	196	22	86	28.6	53	17.3	57	20.1	
Mostly True	94	10.5	39	13.0	25	8.2	30	10.6	
Completely True	81	9.1	41	13.6	17	5.6	23	8.1	
**Men of my race trust medical professionals.**									**<0.001**
Not at all	92	10.3	43	14.3	7	2.3	42	14.8	

Slightly True	168	18.9	80	26.6	30	9.8	58	20.4	
Moderately True	282	31.6	89	29.6	91	29.7	102	35.9	
Mostly True	223	25	54	17.9	112	36.6	57	20.1	
Completely True	126	14.1	35	11.6	66	21.6	25	8.8	
**Men of my race rarely receive quality medical care**									**<0.001**
Not at all	220	24.7	46	15.3	109	35.6	65	22.9	
Slightly True	151	16.9	57	18.9	39	12.7	55	19.4	
Moderately True	236	26.5	91	30.2	61	19.9	84	29.6	
Mostly True	168	18.9	72	23.9	56	18.3	40	14.1	
Completely True	116	13	35	11.6	41	13.4	40	14.1	

Supplementary Table 2: Sociodemographic characteristics of men aged 45 and above (n=435) (Dates of data collection)

	Race and Ethnicity									
	**Total**		**Black**		**White**		**AIAN**		**P-value**
	**No.**	**Col %**	**No.**	**Col %**	**No.**	**Col %**	**No.**	**Col %**	
**Age Group**									**0.6**
45-59	215	49.4	46	46.5	104	52.0	65	47.8	
60-75	220	50.6	53	53.5	96	48.0	71	52.2	
**Census Region**									**0.01**
South	101	23.3	30	30.3	39	19.5	32	23.9	
West	153	35.3	28	28.3	66	33.0	59	44.0	
Northeast	47	10.9	11	11.1	23	11.5	13	9.7	
Midwest	98	22.6	26	26.3	47	23.5	25	18.7	
DC and Puerto Rico	34	7.9	4	4.0	25	12.5	5	3.7	
**Marital Status**									**0.021**
Single	85	19.6	27	27.3	38	19.0	20	14.9	
Married/In a relationship	263	60.7	49	49.5	132	66.0	82	61.2	
Divorced/widowed/separated	85	19.6	23	23.2	30	15.0	32	23.9	
**Sexual Orientation**									**0.227**
Heterosexual	405	93.5	97	98.0	186	93.0	122	91.0	
Homosexual	13	3.0	2	2.0	6	3.0	5	3.7	
Bisexual/Questioning	15	3.5	0	0.0	8	4.0	7	5.2	
**Educational Attainment**									**0.372**
High school or below	90	20.8	19	19.2	37	18.5	34	25.4	
Some college/Associates degree	166	38.3	40	40.4	73	36.5	53	39.6	
Bachelor’s degree and above	177	40.9	40	40.4	90	45.0	47	35.1	

**Health Professional**									**0.428**
No	409	94.5	94	94.9	186	93.0	129	96.3	
Yes	24	5.5	5	5.1	14	7.0	5	3.7	
**Income Category**									**<0.001**
34,999 and below	158	36.6	33	33.7	55	27.5	70	52.2	
35,000-74,999	133	30.8	35	35.7	59	29.5	39	29.1	
75,000 and above	141	32.6	30	30.6	86	43.0	25	18.7	
**Employed**									**0.022**
No	85	19.7	24	24.5	28	14.0	33	24.6	
Yes	347	80.3	74	75.5	172	86.0	101	75.4	
**Insurance Status**									**0.007**
No	47	10.9	7	7.1	16	8.0	24	17.9	
Yes	385	89.1	91	92.9	184	92.0	110	82.1	
**Religious Affiliation**									**<0.001**
Christian	296	68.5	73	74.5	143	71.5	80	59.7	
Muslim	2	0.5	0	0.0	1	0.5	1	0.7	
Atheist	32	7.4	4	4.1	25	12.5	3	2.2	
Other	102	23.6	21	21.4	31	15.5	50	37.3	
**Frequency of Religious Activity Attendance**									**<0.001**
Never	139	32.2	19	19.4	79	39.5	41	30.6	
Occasionally	184	42.6	46	46.9	69	34.5	69	51.5	
Regular	109	25.2	33	33.7	52	26.0	24	17.9	
**Current tobacco smoking frequency**									**0.009**
Daily	96	22.2	15	15.3	38	19.0	43	32.1	

Less than daily	22	5.1	7	7.1	6	3.0	9	6.7	
Not at all	310	71.8	75	76.5	155	77.5	80	59.7	
Don't know	4	0.9	1	1.0	1	0.5	2	1.5	
**Has Regular Provider**									**0.282**
No	69	16.0	14	14.3	28	14.0	27	20.1	
Yes	363	84.0	84	85.7	172	86.0	107	79.9	
**Has Seen Provider in Last 12 mos**									**0.413**
No	85	19.7	16	16.3	38	19.0	31	23.1	
Yes	347	80.3	82	83.7	162	81.0	103	76.9	
**Family History of Cancer**									**0.366**
No	250	57.9	62	63.3	112	56.0	76	56.7	
Yes	153	35.4	31	31.6	77	38.5	45	33.6	
Not sure	29	6.7	5	5.1	11	5.5	13	9.7	
**Family History of CRC**									**0.622**
No	359	83.1	81	82.7	165	82.5	113	84.3	
Yes	33	7.6	10	10.2	16	8.0	7	5.2	
Not sure	40	9.3	7	7.1	19	9.5	14	10.4	
**Ever Diagnosed with CRC**									**0.618**
No	429	99.3	98	100.0	198	99.0	133	99.3	
Yes	3	0.7	0	0.0	2	1.0	1	0.7	
**Did the recent death of Chadwick Boseman increase your awareness of CRC?**									**<0.001**
No	301	69.7	43	43.9	148	74.0	110	82.1	
Yes	131	30.3	55	56.1	52	26.0	24	17.9	

Supplementary Table 3: Masculinity barriers to medical care scale measures by race/ethnicity among respondents 45+ (n=432)

	Total		Black		White		AIAN		
	**No.**	**Col %**	**No.**	**Col %**	**No.**	**Col %**	**No.**	**Col %**	**P-Value**
**As a provider, I assure the needs of my family are met.**									**0.559**
Not at all	10	2.3	1	1.0	5	2.5	4	3.0	
Slightly True	12	2.8	2	2.1	5	2.5	5	3.7	
Moderately True	46	10.7	12	12.4	24	12.0	10	7.5	
Mostly True	96	22.3	16	16.5	50	25.0	30	22.4	
Completely True	267	61.9	66	68.0	116	58.0	85	63.4	
**As a provider, I take risks for my family even if I may get hurt or put myself in danger.**									**0.003**
Not at all	86	20.0	21	21.6	44	22.0	21	15.7	
Slightly True	49	11.4	5	5.2	27	13.5	17	12.7	
Moderately True	92	21.3	21	21.6	47	23.5	24	17.9	
Mostly True	80	18.6	17	17.5	44	22.0	19	14.2	
Completely True	124	28.8	33	34.0	38	19.0	53	39.6	
**As a provider, I have been influenced by male family members to be active in my family's life.**									**0.315**
Not at all	87	20.2	22	22.7	39	19.5	26	19.4	
Slightly True	38	8.8	8	8.2	12	6.0	18	13.4	
Moderately True	87	20.2	19	19.6	42	21.0	26	19.4	
Mostly True	100	23.2	21	21.6	55	27.5	24	17.9	
Completely True	119	27.6	27	27.8	52	26.0	40	29.9	
**As a provider, I get an extra push to succeed for my family when I believe somebody is expecting me to fail.**									**0.015**
Not at all	92	21.3	13	13.4	43	21.5	36	26.9	
Slightly True	42	9.7	7	7.2	21	10.5	14	10.4	
Moderately True	98	22.7	25	25.8	50	25.0	23	17.2	

Mostly True	93	21.6	23	23.7	50	25.0	20	14.9	
**Completely True**	**106**	**24.6**	**29**	**29.9**	**36**	**18.0**	**41**	**30.6**	
**When it comes to my health, I figure problems out on my own.**									**0.006**
Not at all	56	13.0	22	22.7	19	9.5	15	11.2	
Slightly True	80	18.6	13	13.4	41	20.5	26	19.4	
Moderately True	134	31.1	27	27.8	74	37.0	33	24.6	
Mostly True	110	25.5	22	22.7	50	25.0	38	28.4	
Completely True	51	11.8	13	13.4	16	8.0	22	16.4	
**When it comes to my health, I rely on the opinions of others.**									**0.097**
Not at all	106	24.6	33	34.0	38	19.0	35	26.1	
Slightly True	104	24.1	22	22.7	47	23.5	35	26.1	
Moderately True	124	28.8	19	19.6	70	35.0	35	26.1	
Mostly True	64	14.8	17	17.5	28	14.0	19	14.2	
Completely True	33	7.7	6	6.2	17	8.5	10	7.5	
**When it comes to my health, I make my own decisions.**									**0.435**
Not at all	11	2.6	5	5.2	4	2.0	2	1.5	
Slightly True	30	7.0	9	9.3	14	7.0	7	5.2	
Moderately True	93	21.6	21	21.6	45	22.5	27	20.1	
Mostly True	136	31.6	29	29.9	68	34.0	39	29.1	
Completely True	161	37.4	33	34.0	69	34.5	59	44.0	
**When it comes to my health, I rely on remedies that others suggest to me.**									**0.013**
Not at all	121	28.1	36	37.1	49	24.5	36	26.9	
Slightly True	126	29.2	28	28.9	53	26.5	45	33.6	
Moderately True	103	23.9	11	11.3	59	29.5	33	24.6	
Mostly True	59	13.7	13	13.4	30	15.0	16	11.9	
Completely True	22	5.1	9	9.3	9	4.5	4	3.0	

**A problem with my health would be a big deal.**									**<0.001**
Not at all	17	3.9	2	2.1	4	2.0	11	8.2	
Slightly True	45	10.4	6	6.2	19	9.5	20	14.9	
Moderately True	110	25.5	17	17.5	55	27.5	38	28.4	
Mostly True	118	27.4	23	23.7	62	31.0	33	24.6	
Completely True	141	32.7	49	50.5	60	30.0	32	23.9	
**I wait until I am sure a problem with my health is serious.**									**0.011**
Not at all	105	24.4	35	36.1	37	18.5	33	24.6	
Slightly True	89	20.6	18	18.6	42	21.0	29	21.6	
Moderately True	116	26.9	23	23.7	55	27.5	38	28.4	
Mostly True	87	20.2	13	13.4	54	27.0	20	14.9	
Completely True	34	7.9	8	8.2	12	6.0	14	10.4	
**I take action immediately when I have a health problem.**									**0.013**
Not at all	35	8.1	5	5.2	16	8.0	14	10.4	
Slightly True	94	21.8	14	14.4	50	25.0	30	22.4	
Moderately True	103	23.9	20	20.6	59	29.5	24	17.9	
Mostly True	94	21.8	24	24.7	39	19.5	31	23.1	
Completely True	105	24.4	34	35.1	36	18.0	35	26.1	
**A problem with my health will go away on its own.**									**0.183**
Not at all	209	48.5	54	55.7	83	41.5	72	53.7	
Slightly True	98	22.7	19	19.6	52	26.0	27	20.1	
Moderately True	85	19.7	14	14.4	48	24.0	23	17.2	
Mostly True	32	7.4	7	7.2	14	7.0	11	8.2	
Completely True	7	1.6	3	3.1	3	1.5	1	0.7	
**It is important to talk to others about my feelings.**									**0.199**
Not at all	65	15.1	9	9.3	29	14.5	27	20.1	
Slightly True	88	20.4	15	15.5	49	24.5	24	17.9	

Moderately True	138	32.0	37	38.1	58	29.0	43	32.1	
Mostly True	78	18.1	18	18.6	37	18.5	23	17.2	
Completely True	62	14.4	18	18.6	27	13.5	17	12.7	
**I believe in remaining strong at all times.**									**0.223**
Not at all	7	1.6	0	0.0	4	2.0	3	2.2	
Slightly True	35	8.1	10	10.3	20	10.0	5	3.7	
Moderately True	103	23.9	21	21.6	52	26.0	30	22.4	
Mostly True	147	34.1	29	29.9	68	34.0	50	37.3	
Completely True	139	32.3	37	38.1	56	28.0	46	34.3	
**Acknowledging my own emotions is always helpful.**									**0.003**
Not at all	25	5.8	2	2.1	10	5.0	13	9.7	
Slightly True	67	15.5	7	7.2	34	17.0	26	19.4	
Moderately True	111	25.8	26	26.8	61	30.5	24	17.9	
Mostly True	127	29.5	35	36.1	58	29.0	34	25.4	
Completely True	101	23.4	27	27.8	37	18.5	37	27.6	
**Physical pain in my body would never lead me to show any emotions.**									**0.001**
Not at all	120	27.8	33	34.0	64	32.0	23	17.2	
Slightly True	101	23.4	19	19.6	49	24.5	33	24.6	
Moderately True	110	25.5	25	25.8	56	28.0	29	21.6	
Mostly True	66	15.3	12	12.4	23	11.5	31	23.1	
Completely True	34	7.9	8	8.2	8	4.0	18	13.4	
**Having medical exams below the waist would disrespect my sexuality.**									**0.307**
Not at all	351	81.4	76	78.4	162	81.0	113	84.3	
Slightly True	25	5.8	3	3.1	14	7.0	8	6	
Moderately True	36	8.4	9	9.3	18	9.0	9	6.7	
Mostly True	15	3.5	7	7.2	5	2.5	3	2.2	

Completely True	4	0.9	2	2.1	1	0.5	1	0.7	
**Medical professionals touching me below the waist is fine when it relates to my health.**									**0.324**
Not at all	20	4.6	7	7.2	8	4.0	5	3.7	
Slightly True	22	5.1	5	5.2	8	4.0	9	6.7	
Moderately True	50	11.6	14	14.4	26	13.0	10	7.5	
Mostly True	89	20.6	19	19.6	47	23.5	23	17.2	
Completely True	250	58	52	53.6	111	55.5	87	64.9	
**Health problems below the waist would be embarrassing to me.**									**0.045**
Not at all	174	40.4	44	45.4	65	32.5	65	48.5	
Slightly True	90	20.9	20	20.6	43	21.5	27	20.1	
Moderately True	84	19.5	18	18.6	47	23.5	19	14.2	
Mostly True	51	11.8	11	11.3	30	15.0	10	7.5	
Completely True	32	7.4	4	4.1	15	7.5	13	9.7	
**I would be comfortable with others knowing about health problems below the waist.**									**0.994**
Not at all	119	27.6	29	29.9	56	28.0	34	25.4	
Slightly True	82	19	18	18.6	40	20.0	24	17.9	
Moderately True	104	24.1	21	21.6	49	24.5	34	25.4	
Mostly True	65	15.1	15	15.5	28	14.0	22	16.4	
Completely True	61	14.2	14	14.4	27	13.5	20	14.9	
**Medical professionals want men of my race to live a long time.**									**0.002**
Not at all	30	7	9	9.3	9	4.5	12	9	
Slightly True	39	9	15	15.5	9	4.5	15	11.2	
Moderately True	110	25.5	27	27.8	54	27.0	29	21.6	
Mostly True	126	29.2	29	29.9	54	27.0	43	32.1	
Completely True	126	29.2	17	17.5	74	37.0	35	26.1	

**Men of my race are treated like guinea pigs by medical professionals.**								**<0.001**
Not at all	214	49.7	23	23.7	129	64.5	62	46.3	
Slightly True	84	19.5	26	26.8	28	14.0	30	22.4	
Moderately True	82	19	29	29.9	27	13.5	26	19.4	
Mostly True	27	6.3	9	9.3	10	5.0	8	6	
Completely True	24	5.6	10	10.3	6	3.0	8	6	
**Men of my race trust medical professionals.**									**<0.001**
Not at all	34	7.9	11	11.3	5	2.5	18	13.4	
Slightly True	73	16.9	30	30.9	17	8.5	26	19.4	
Moderately True	129	29.9	27	27.8	59	29.5	43	32.1	
Mostly True	138	32	23	23.7	76	38.0	39	29.1	
Completely True	57	13.2	6	6.2	43	21.5	8	6	
**Men of my race rarely receive quality medical care.**									**<0.001**
Not at all	137	31.8	16	16.5	87	43.5	34	25.4	
Slightly True	74	17.2	22	22.7	22	11.0	30	22.4	
Moderately True	95	22	28	28.9	32	16.0	35	26.1	
Mostly True	74	17.2	21	21.6	34	17.0	19	14.2	
Completely True	51	11.8	10	10.3	25	12.5	16	11.9	

Supplementary Table 4: Associations of masculinity barriers to health care with intention to screen for CRC among racial ethnic minority men aged 45 years and above

	Black Adult Men			White Adult Men			AIAN Adult Men		
	**OR**	**95% CI**		**OR**	**95% CI**		**OR**	**95% CI**	
**Overall Scale Score**	**1.76**	**0.57**	**5.45**	**0.50**	**0.28**	**1.05**	**0.34**	**0.14**	**0.84**
Provider Role	0.68	0.25	1.84	0.71	0.38	1.33	1.04	0.46	2.35
Health-Related Self-Reliance	1.85	0.66	5.13	1.82	1.00	3.35	0.88	0.38	2.02
Health Problem Minimization	0.93	0.31	2.74	0.45	0.24	0.85	0.39	0.15	1.00
Restrictive Emotionality	0.44	0.24	0.82	0.44	0.24	0.82	0.45	0.19	1.04
Fear of being Perceived as Gay	2.28	0.76	6.79	0.76	0.41	1.41	1.06	0.44	2.58
Medical Mistrust	2.18	0.80	5.95	0.32	0.16	0.66	1.12	0.47	2.65

Adjusted for age, marital status, educational attainment, insurance status, regular provider, family history of CRC
